# Mechanistic and Therapeutic Implications of Extracellular Vesicles as a Potential Link Between Covid-19 and Cardiovascular Disease Manifestations

**DOI:** 10.3389/fcell.2021.640723

**Published:** 2021-02-11

**Authors:** Gianluigi Pironti, Daniel C. Andersson, Lars H. Lund

**Affiliations:** ^1^Department of Medicine, Cardiology Research Unit, Karolinska Institutet, Stockholm, Sweden; ^2^Department of Physiology and Pharmacology, Karolinska Institutet, Stockholm, Sweden; ^3^Heart, Vascular and Neurology Theme, Unit of Cardiology, Karolinska University Hospital, Stockholm, Sweden

**Keywords:** extracellular vescicles, COVID–19, renin—angiotensin—aldosterone system, cardiovascular disease, MSC derived exosomes, cardiosphere-derived cell, exosomes, cardiomyopathy

## Abstract

Extracellular vesicles (EVs), which are cell released double layered membrane particles, have been found in every circulating body fluid, and provide a tool for conveying diverse information between cells, influencing both physiological and pathological conditions. Viruses can hijack the EVs secretory pathway to exit infected cells and use EVs endocytic routes to enter uninfected cells, suggesting that EVs and viruses can share common cell entry and biogenesis mechanisms. SARS-CoV-2 is responsible of the coronavirus disease 2019 (Covid-19), which may be accompanied by severe multi-organ manifestations. EVs may contribute to virus spreading via transfer of virus docking receptors such as CD9 and ACE2. Covid-19 is known to affect the renin angiotensin system (RAS), and could promote secretion of harmful EVs. In this scenario EVs might be linked to cardiovascular manifestations of the Covid-19 disease through unbalance in RAS. In contrast EVs derived from mesenchymal stem cells or cardiosphere derived cells, may promote cardiovascular function due to their beneficial effect on angiogenesis, fibrosis, contractility and immuno-modulation. In this article we assessed the potential impact of EVs in cardiovascular manifestations of Covid-19 and highlight potential strategies to control the extracellular signaling for future therapies.

## Introduction

Cells are continuously secreting extracellular vesicles (EVs) that include large apoptotic bodies (1–5 μm), microvesicles (100–1,000 nm) or small exosomes (30–100 nm), which vary in their abundance, size, and composition. Enclosed by a lipid bilayer, EVs are thus very stable and convey cytoplasmic molecules including proteins, RNAs, lipids, and metabolites (Robbins and Morelli, 2014).

Exosomes originate from processing of early endosomes by endosomal sorting complex to form intraluminal vesicles within larger multivesicular bodies (MVB) that traffic in the cytoplasm (Thery et al., 2002). The fusion of MVB to the plasma membrane allows the release of their intraluminal vesicles (now called exosomes) into the extracellular microenvironment (Thery et al., 2002). Initially these vesicles were thought to be secreted by cells merely to eliminate obsolete molecules, but the importance of exosomes in conveying cellular information, with potent autocrine and paracrine biological activities, is now well known as they are released by many different cell types and can be found in most bodily fluids (van Niel et al., 2018).

Microvesicles have size overlap and share some surface markers, such as CD9 and CD63 (Crescitelli et al., 2013; Andreu and Yanez-Mo, 2014) making even more challenging the distinction from exosomes, thus they both are often referred as extracellular vesicles. However, microvesicles have a distinctive biogenesis originating from outward budding of plasma membrane (Kalra et al., 2016). The apoptotic bodies represent the largest EVs and are secreted by cells undergoing programmed cell death, by membrane blebbing and bulging, promoting the clearance of apoptotic material. The intrinsic capacity of EVs in conveying biologic material between proximal and distant cells have been used for therapeutic applications in order to facilitate drug delivery, but it is also used by pathogens to facilitate infection spreading and cause multi-organ diseases. This aspect should be taken in consideration in the perspective of engineering EVs with implemented delivery capacity. In this review we use the term EVs to refer to the two categories microvesicles and exosomes.

## Can EVs Promote SARS-CoV-2 Virus Spreading?

Many viruses, including coronaviruses, are known to enter the EVs avenue during synthesis and intra-host spreading, in a caveolin-1-dynamin-dependent mechanism (Owczarek et al., 2018; Badierah et al., 2020). Whether the SARS-CoV-2 virus, which causes the coronavirus disease 2019 (Covid-19) is involved in EVs trafficking is still unknown. However, *in vitro* data show that alveolar cells infected with SARS-CoV-1 present whole virions in the secretory vesicles near the plasma membrane of infected cells (Qian et al., 2013; Mason, 2020). Whether SARS-CoV-2 virions also can accumulate in secreting vesicles has not been validated yet, although electron microscopy images of lung biopsy in autopsy specimen from deceased Covid-19 patient intriguingly show SARS-CoV-2 virion within double membrane vesicles (Dittmayer et al., 2020). Moreover, histopathological analysis of renal specimen from deceased patients with Covid-19 showed that SARS-CoV-2 virions are mostly found in the cytoplasm and some vacuoles containing assembling virions similar to what has been seen with SARS-CoV-1 (Farkash et al., 2020; Su et al., 2020). Due to the size of coronavirus (60–140 nm) it is possible that bigger EVs such as microparticles might mediate whole virion transport, while exosomes might carry viral fragments. During an infection, big apoptotic bodies released from infected/injured cells might be an easy way to carry virions due to the larger size, however apoptotic bodies present surface bridging molecules that triggers phagocytosis, which could contribute to contain rather than spreading SARS-CoV-2 in the body (Arandjelovic and Ravichandran, 2015). However, exosomes and microparticles contain virus receptors that make EVs recipient cells susceptible to virus entry (Earnest et al., 2015). Indeed tetraspanin protein CD9 expressed by EVs formed cell membrane complex of the co-receptor dipeptidyl peptidase 4 (DPP4) and the type II transmembrane serine protease member TMPRSS2, a CoV-activating protease, facilitating proteolytic priming events with cells for MERS-CoV pseudovirus, which is necessary for virus cell entry and can be blocked by CD9 inhibition (Earnest et al., 2015). Similarly, SARS-CoV-2 uses DPP4 (Li Y. et al., 2020; Solerte et al., 2020) and TMPRSS2 (Hoffmann et al., 2020) to facilitate cell entry. Although it might be reasonable to think that also SARS-CoV-2 virulence relies on CD9 activity by clustering and scaffolding receptors and proteases for efficient cell entry, this hypothesis has not been validated yet in current literature.

Thus, coronavirus such as SARS-CoV-1 and MERS-CoV may be directed into the exosomal pathway or released from microvesicles, upon intracellular entry, and its components, such as mRNAs and proteins, including spike protein (Kuate et al., 2007), are packaged into EVs for secretion (Hassanpour et al., 2020).

Although conventional biochemistry techniques allow the detection of viral components such as viral RNA or spike proteins, there is currently no empirical evidence that EVs secreted from infected cells contain specific fragments of SARS-CoV-2. EM images potentially would be a method to investigate this, but purification techniques available at the moment cannot separate virions from EVs due to their overlapping size. Therefore, the isolation procedures need to be implemented in order to identify specific viral components (RNA or proteins) carried by EVs. Whether EVs released from infected cells have harmful or protective roles is controversial: vesicles containing only viral fragments might stimulate the immune system for their antigen priming activity, while the whole virion hidden in bigger vesicles could potentially elicit viral dissemination by escaping the immune recognition and further spread the virus within the body.

In addition to CD9, it has been shown that exosomes can transfer the angiotensin converting enzyme 2 (ACE2) receptor (Wang et al., 2020), which SARS-CoV-2 uses for cell entry. It is reasonable to believe that increased levels of ACE2 might promote more virus entry similarly to what has been observed in cellular models transfected with human ACE2 (Blanco-Melo et al., 2020; Ou et al., 2020). Alternatively EVs containing ACE2 might be used to decoy the SARS-CoV-2 and limit virus spreading (Inal, 2020). The presence of docking station for virus priming such as ACE2 is necessary but not sufficient for virus entry as the proteases activity of TMPRSS2 is critical for fusion and internalization of ACE-2/SARS-CoV2 spike complex (Hoffmann et al., 2020), and co-receptors such as neuropilin-1 (NRP-1) can significantly potentiate SARS-CoV-2 infectivity (Cantuti-Castelvetri et al., 2020). Both NRP-1 and TMPRSS2 have been found in EVs (Munshi et al., 2019; Chi et al., 2020). As the inter-cellular trafficking pathways of viruses and EVs are similar or shared, pharmacological inhibition of EVs trafficking has been proposed as an important antiviral approach in order to limit the systemic spreading of SARS-CoV-2 (Urciuoli and Peruzzi, 2020).

It has been reported that during SARS-CoV-1 and MERS-CoV infections there were increased levels of circulating exosomes enriched in lung-associated self-antigens as well as viral antigens (Gunasekaran et al., 2020). Thus, it might be possible that SARS-CoV-2 infected cells increase the production of exosomes enriched in viral components. In this way, exosomes containing pathogenic proteins and RNA released from infected cells may induce host humoral and cellular immune response (Gunasekaran et al., 2017) and could potentially play anti-infective roles (Zhang et al., 2018). In contrast, it is conceivable that viral material might also be hidden within EVs vesicles using a sort of “trojan horse” strategy for immune system evasion, which might potentially be linked to the variable detection of viral RNA in testing patients with ongoing, or recovered, Covid-19 (Elrashdy et al., 2020). Although different reasons (including methodological problems) may be underlying false negative PCR tests for viral detection, it is intriguing to speculate that EVs could be a hiding place for viral material.

## How Do EVs Promote the Interplay Between the Renin-Angiotensin System (RAS) and SARS-CoV-2?

In the progression of chronic heart failure, compensatory but maladaptive renin-angiotensin system (RAS) activation promotes cardiac remodeling (Zhou et al., 2016). Current literature describes a modulatory role of EVs in RAS through intercellular trafficking by exosomes. It has been shown that increased angiotensin type II (AngII) levels in heart failure, elicit secretion of exosomes by cardiac fibroblasts, which induces neighboring cardiomyocytes to release more AngII and express AngII receptors (AT_1_R and AT_2_R) through a feedback process that promotes myocardial hypertrophy (Lyu et al., 2015). Moreover, increased AngII in plasma and cardiac pressure overload leads to release of exosomes enriched in AT_1_R, which target resistance vessels and cardiomyocytes, aggravating cardiac remodeling (Pironti et al., 2015). Post-mortem autopsy analysis showed that almost 35% of patients who succumbed to SARS-CoV-1 (in 2003) presented detectable viral SARS-CoV-1 genome in heart samples, and this was associated with myocardial fibrosis, inflammation, and reduced myocardial ACE2 expression (Oudit et al., 2009). SARS-CoV-2 viral particles have been found in cardiac biopsies of Covid-19 patients (Dolhnikoff et al., 2020; Tavazzi et al., 2020), and it has been reported that this virus can infect human cardiomyocytes and induce cardiotoxicity (Bojkova et al., 2020).

Both SARS-CoV-1 and SARS-CoV-2 uses ACE2 as docking station to infect target cells and the internalization of virus/enzyme complex leads to loss of enzymatically active ACE2 at the cell surface (Gheblawi et al., 2020; Hoffmann et al., 2020).

As RAS-inhibitors can upregulate expression of ACE2, there were initial concerns that use of RAS-antagonists may increase risk or severity of Covid-19 as more docking stations would theoretically be present. However, the use of RAS inhibitor therapy was not associated with increased infectivity or severity of disease (Li J. et al., 2020; Zhang et al., 2020).

**FIGURE 1:**
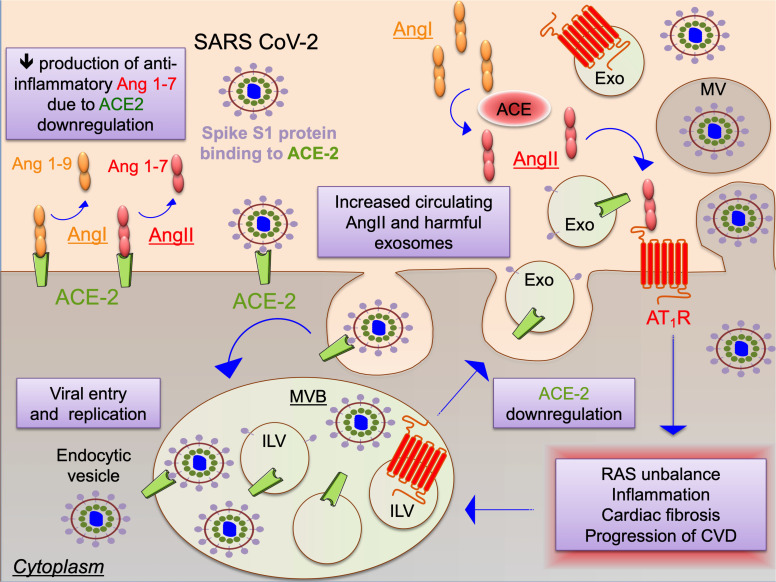
Schematic representation of the potential interplay between the renin-angiotensin system (RAS) and SARS-CoV-2 as mediated by extracellular vesicles. SARS coronaviruses use spike protein S1 to bind ACE-2 as docking station leading to cell entry and also to ACE2 down-regulation. Increased levels of angiotensin type II (AngII) aggravate RAS, promoting inflammation, cardiac fibrosis and progression of cardiovascular diseases (CVD). SARS-CoV-2 infection and RAS trigger the endocytic machinery, leading to internalization of ACE2, virion components and angiotentin II type 1 receptor (AT_1_R) into intraluminal vesicles (ILV) within multivesicular bodies (MVB), which are released as exosomes (Exo) upon fusion of the MVB with the cell plasma membrane. SARS-CoV-2 spreading may be enhanced also through release of virions in larger microvesicles (MV) upon plasma membrane budding of infected cells. Exosomes or other EVs might be responsible for manifestations and progression of cardiovascular disease in Covid-19 through maladaptive RAS signaling.

As cells are infected by SARS-CoV-2, the ACE2 expression is down-regulated due to virus/enzyme complex internalization. The reduction of ACE2 enzymatic activity results in an imbalance within the RAS, which stimulates neutrophil infiltration, unopposed AngII accumulation and may promote acute lung injury. Thus, RAS-inhibitors may also be protective. Less attention has been given to the fact that increased circulating AngII levels could elicit the endocytosis of ACE-2 bound to SARS-CoV-2 via a AT1R-dependent mechanism (Offringa et al., 2020), which may promote ACE2 degradation (Deshotels et al., 2014). Furthermore, reduced expression levels of ACE2 in the heart, following SARS-CoV-2 infection, may impair the conversion of AngII to the Ang1-7 heptapeptide, which is anti-inflammatory, anti-fibrotic, and cardioprotective (Patel et al., 2014) and represents the main mediators of the protective RAS signaling activating via AT2R and MasR (Santos et al., 2003). Thus, in addition to downregulating ACE2 and Ang1-7, unbalanced RAS activation through exosomes during Covid-19 may represent a novel detrimental pathway that could be responsible for some of the cardiovascular disease manifestations in Covid-19 (Figure 1). Indeed in a yet ongoing promising clinical trial, a non-peptide AT_2_R agonist is used as therapy to counterbalance the potentially harmful RAS effects during Covid-19 (Angiotensin II Type Two Receptor Agonist in COVID-19 Trial ATTRACT study, NCT04452435) (Steckelings and Sumners, 2020).

Although EVs release as results of RAS disturbance during SARS-CoV-2 infection has not yet been demonstrated, we here provide a provocative hypothesis of a link between EVs, Covid-19 and RAS based on two demonstrated evidences: (i) SARS-CoV-2 infection causes disturbance of RAS; (ii) RAS-dependent release of exosomes in e.g., hypertension are known to aggravate cardiovascular diseases and myocardial remodeling.

## How to Combat the Cytokine Storm During Covid-19 Using EVs as Therapy?

Severe Covid-19 display a systemic pro-inflammatory state often referred to as a cytokine storm with critical illness and manifestations such as acute respiratory distress syndrome (ARDS), pulmonary edemea, thromboembolism, and acute cardiac injury, which come with high mortality (Huang et al., 2020; Leisman et al., 2020).

Mesenchymal stem cells (MSCs) have powerful immuno-modulatory effects and are considered potentially effective therapeutic assets to combat a wide spectrum of inflammatory-driven diseases but also clinically useful in preventing inflammatory activation from acute graft vs. host disease following allogenic hematopoietic stem-cell transplantation (Hashmi et al., 2016; Bazzoni et al., 2020). Thus, MSCs might be used to prevent or reduce the cytokine storm caused by SARS-CoV-2. Interestingly, EVs released from MSCs is one of the mechanisms whereby MSCs may promote anti-inflammatory effects (Hashmi et al., 2016; Bazzoni et al., 2020). Indeed, several studies have described beneficial effect of MSCs-derived EVs following myocardial injury by increasing angiogenesis (Liang et al., 2016; Gong et al., 2017) improving cardiac contractility (Kang et al., 2015; Ma et al., 2017; Mayourian et al., 2017). Moreover stem cells derived from cardiac tissues, i.e., cardiosphere derived cells (CDC), produce EVs able to reduce inflammation and fibrosis signaling (Gray et al., 2015; Gallet et al., 2017; Rogers et al., 2020). The intercellular communication between macrophages, fibroblasts and endothelial cells orchestrates the progression of chronic heart failure and elicit myocardial fibrosis. Thus, in the context of anti-fibrogenesis, EVs may have a beneficial effect by promoting the trans-differentiation of macrophages from pro-inflammatory M1 into M2-like cells with immunosuppressive phenotype (Silva et al., 2017). This process is important for the resolution of inflammation and might be particularly relevant in the context of therapy meant to combat the systemic pro-inflammatory state in Covid-19.

Interferon gamma (INF-γ) is a major proinflammatory cytokine secreted by activated T cells and natural killer cells, which promotes macrophage activation and mediate host defense against pathogen infection. Decreased plasma levels of INF-γ in the early stage of the disease (before the onset of cytokine storm) were associated with increased risk of pulmonary fibrosis in Covid-19 patients (Hu et al., 2020). Indeed INF-γ has antiviral action, through enhancing the viral antigen processing and presentation to cytotoxic T lymphocytes for virus clearance, and has also anti-fibrotic effect. Thus early intervention of anti-viral infection using IFN-γ could be significant in the inhibition of fibrosis for better functional recovery (Hu et al., 2020). Stem cells exposed to proinflammatory cytokine signaling released EVs enriched in mRNA coding for INF-γ signaling pathway, such as STAT1, JAK 1-2 (Cossetti et al., 2014). Interestingly EVs can recycle and deliver free INF-γ and by that, promoting the continuation of pro-inflammatory signaling response in target cells (Cossetti et al., 2014).

Therapeutic approaches for Covid-19 based on MSCs-derived EVs aimed to enhancing the healing process following lung injury, have been taken in consideration (Akbari and Rezaie, 2020; Al-Khawaga and Abdelalim, 2020; Pinky et al., 2020; Tsuchiya et al., 2020). However, experimental evidences of their mechanism of action, and whether MSCs-derived EVs therapy can ameliorate cardiovascular manifestations have not been elucidated. Interestingly, the first clinical trial using MSC derived exosomes that were intravenously injected in Covid-19 patients with moderate-to-severe symptoms (Sengupta et al., 2020) (NCT04276987) showed positive results in terms of safety profile, capacity to restore oxygenation, downregulation of cytokines, and reconstitute immunity. In another clinical trial (NCT04276987), inhalation of aerosol enriched in allogenic EVs derived from adipose MSC have been tested for safety and efficacy in Covid-19 patients with severe symptoms (Pocsfalvi et al., 2020).

The timing of an intervention seems to be a critical aspect in Covid-19 disease. The acute phase of Covid-19 illness is characterized by three pathological phases: (i) the early infection phase when SARS-CoV-2 infiltrates and replicate; (ii) the pulmonary phase characterized by respiratory dysfunction; (iii) finally the hyperinflammation phase driven by the host immunity with an exaggerate inflammatory response (Akhmerov and Marban, 2020). The specific stage of disease must be taken into consideration when commencing different therapeutic interventions e.g., with antiviral, immunopotentiating or anti-inflammatory properties (de Simone and Mancusi, 2020). Although each phase can be defined based on clinical and laboratory findings the preciseness of this diagnosing is not complete thus providing a major challenge for timing of intervention. A too early anti-inflammatory intervention might hinder the viral elimination by host immunity, while a too late anti-inflammatory therapy might have a slight efficacy.

Although EVs containing viral components might elicit the adaptive immune response by presenting the antigen in a process similar to a vaccination, EVs secreted from infected/injured cells could activate macrophage secreting cytokines and contribute to the cytokine storm formation during Covid-19 (Perez et al., 2019). Indeed, EVs can carry pathogen-associated molecular patterns (PAMPs) released from stressed or injured cells and therefore triggering inflammation upon interaction with innate immune cells contributing to inflammation induction and persistence (Bhatnagar et al., 2007). Additionally, EVs containing cytokines (Pizzirani et al., 2007), enzymes involved in the biosynthesis of lipid mediators (Cossetti et al., 2014) and other chemotactic signals (Esser et al., 2010; Kriebel et al., 2018) can contribute to the propagation of inflammation. Interestingly, ACE2 enriched EVs derived from engineered MSC has been considered as a possible Covid-19 therapy in order to decoy the SARS-CoV-2 and limit virus spreading (Inal, 2020).

Thus, a therapeutic application of EVs for Covid-19 must include donor cells with anti-inflammatory activity such as mesenchymal stem cells or cardiosphere derived cells. The anti-inflammatory activity of EVs indicate that this platform can work well in xenogenic applications (de Couto et al., 2017; Gallet et al., 2017), which could simplify the large scale production of EVs for therapeutic applications by using MSCs or CDC derived from large animals rather than humans.

## Conclusion

We have discussed the possible function of EVs in Covid-19 and highlight potential therapeutic application for EVs in modulating the virus infection, the pro-inflammatory state and cardiovascular disease in Covid-19. EVs, such as exosomes could be involved in aggravated cardiovascular manifestations of Covid-19 either by promoting viral docking in cardiac cells or shifting RAS to promote inflammation, coagulation, fibrosis and endothelial dysfunction. Furthermore, EVs derived from engineered MSCs or CDC used as drug delivery system to modulate the cytokine storm of virus spreading represent a promising strategy to combat Covid-19. In summary, understanding the molecular mechanisms behind the entry, replication, and spreading of SARS-CoV-2, in conjunction to EVs trafficking, may provide tools to limit the manifestation of Covid-19.

## Author Contributions

GP substantially contributed to the conception and design of the perspective article, literature search, drafting the article and revising the article critically for important intellectual content. DA and LL contributed in literature search, drafting the article and revising the article critically for important intellectual content. All the authors approved the final version of the article to be published.

## Conflict of Interest

The authors declare that the research was conducted in the absence of any commercial or financial relationships that could be construed as a potential conflict of interest.
